# Supplementary material for: Association between oral microbiome and seven types of cancers in East Asian population: a two-sample Mendelian randomization analysis
Source: Front Mol Biosci. 2023 Nov 21;10:1327893. doi: 10.3389/fmolb.2023.1327893 (PMC10702768; doi:10.3389/fmolb.2023.1327893)
Supplement: Supplementary file 1 [file DataSheet1.ZIP › Supplementary Materials/MR plots of saliva/saliva═╝/colorectal cancer/pheno.1269_to_colorectal cancer_leave_one_out.pdf]

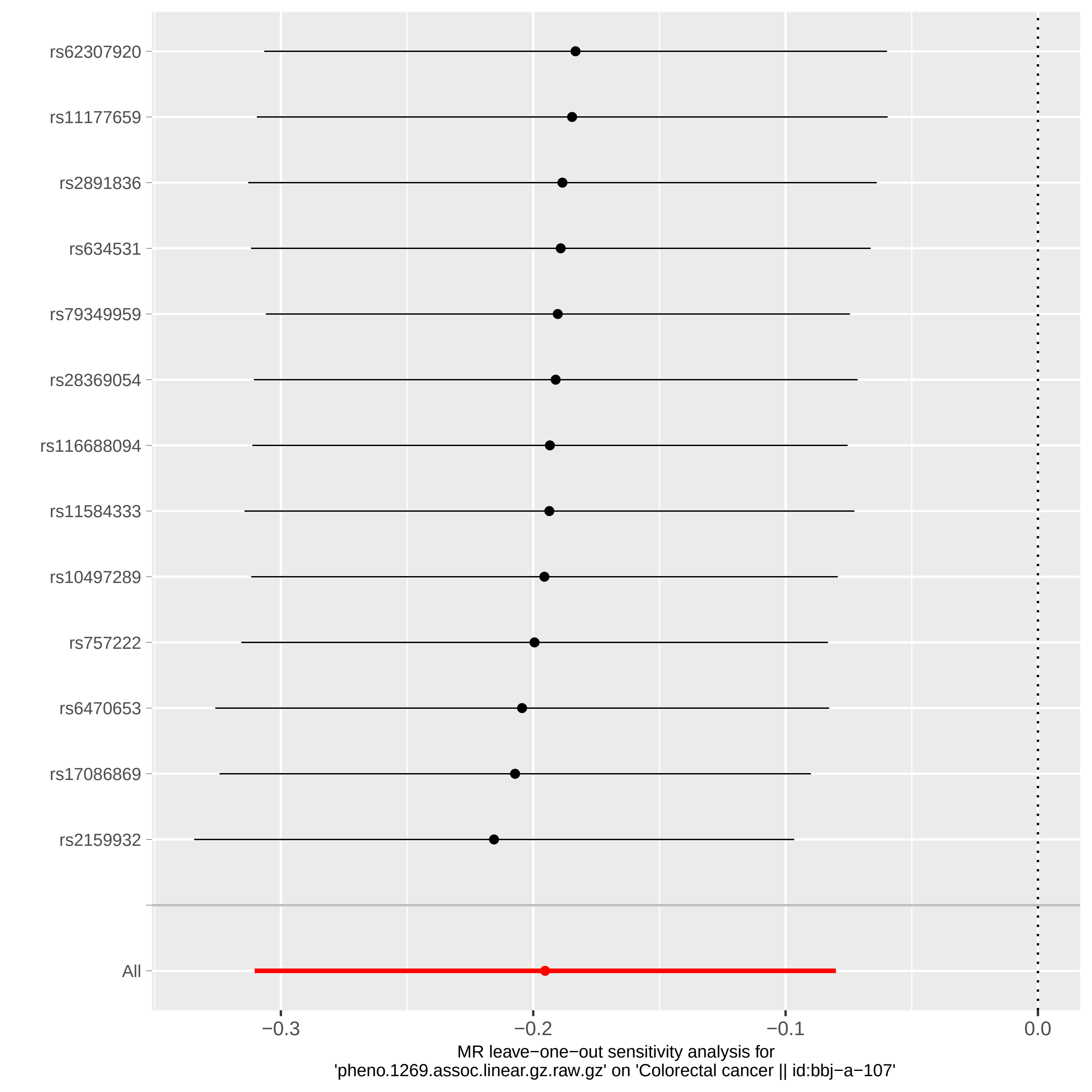
rs62307920
rs11177659
rs2891836
rs634531
rs79349959
rs28369054
rs116688094
rs11584333
rs10497289
rs757222
rs6470653
rs17086869
rs2159932
All
−0.3
−0.2
−0.1
0.0
MR leave−one−out sensitivity analysis for
'pheno.1269.assoc.linear.gz.raw.gz' on 'Colorectal cancer || id:bbj−a−107'
